# Environmental Impact of Fluoroquinolones and Their Photocatalytic Transformation Products: Degradation with Activated Sludge and in Surface Waters, Change in Antimicrobial Activity and Ecotoxicity

**DOI:** 10.3390/ijms27052099

**Published:** 2026-02-24

**Authors:** Wojciech Baran, Daria Madej-Knysak, Oliver Klink, Ewa Adamek

**Affiliations:** 1 Department of General and Analytical Chemistry, Medical University of Silesia, Jagiellońska 4, 41-200 Sosnowiec, Poland; wbaran@sum.edu.pl (W.B.); dmadej@sum.edu.pl (D.M.-K.); 2 Students’ Scientific Society at Department of General and Analytical Chemistry, Medical University of Silesia, Jagiellońska 4, 41-200 Sosnowiec, Poland

**Keywords:** fluoroquinolones, antimicrobial activity, biodegradation, ecotoxicity, activated sludge, intermediates

## Abstract

Fluoroquinolone antibiotics are widely used in medicine and in veterinary medicine. Due to their stable chemical structure, their residues have been detected in the aquatic environment. Photocatalytic degradation is one of the promising methods for the removal of antibiotics, but the formed organic by-products can exhibit antimicrobial activity and still be toxic to aquatic organisms. The purpose of this study was to identify intermediates formed during norfloxacin and enrofloxacin photocatalysis with TiO_2_ and to evaluate in vitro their ecotoxicity towards selected bacteria strains and, in silico, towards algae, daphnids, and fish. Furthermore, changes in the ecotoxicity and composition of solutions after photocatalysis were studied in activated sludge and samples from various natural aquatic ecosystems. After degradation, 24 and 27 intermediates of norfloxacin and enrofloxacin, respectively, were identified, and most of them retained a preserved biologically active pharmacophore. Some intermediates were persistent in the natural environment and may pose long-term ecological risks. In the presence of activated sludge, the majority of fluoroquinolones, as well as most of the intermediates, were degraded. This indicates that the combination of photocatalysis and biological treatment significantly reduces the ecotoxicity of solutions containing fluoroquinolone residues. One of the toxic degradation products was resistant to biodegradation under the conditions used.

## 1. Introduction

**Figure 1:**
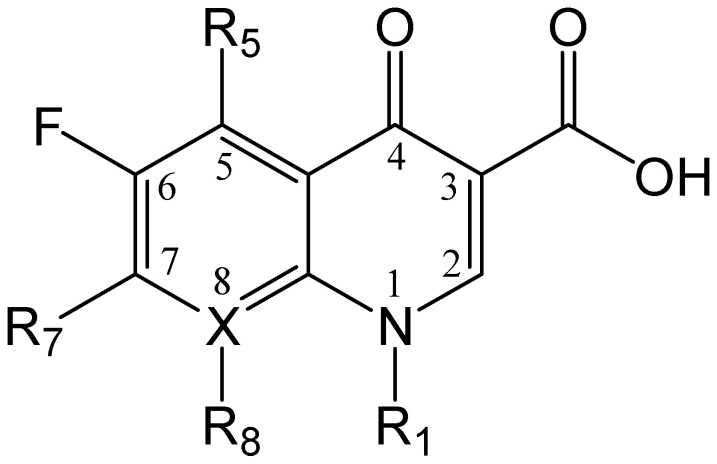
Basic structure of FQs.

Antibiotic residues introduced into surface waters pose an environmental problem due to several significant issues. Their presence disrupts microbiocenoses, promotes the selection of antibiotic-resistant bacteria, and disrupts biological processes important for ecosystem balance. Long-term consequences include a reduction in biodiversity and potential negative side effects on public health, such as the reduced effectiveness of antibiotic therapies [1,2,3,4,5,6,7]. A group of antibiotics considered particularly dangerous consists of fluoroquinolones (FQs). They are widely used to treat infections caused by bacteria that are resistant to other groups of antibiotics. The general structure of the FQ molecule is shown in Figure 1.

The elements necessary for the antibacterial action of FQs (the pharmacophore) include the nitrogen atom N1 in the pyridine ring, the double bond between the atoms C2 and C3, the carboxyl group at C3, the carbonyl group at C4, and the fluorine atom at C6. The presence and type of the R1, R5, R7, and R8 substituents improve the antibacterial activity of FQs.

FQs act by binding to DNA gyrase-DNA and topoisomerase IV-DNA complexes, followed by a rapid and reversible inhibition of DNA replication. This leads to chromosome fragmentation, induction of the SOS response, and rapid bacterial cell death [8,9].

The main sources of FQs in the environment are waste from livestock and poultry farming, pharmaceutical industry waste, and municipal waste. According to the study by Larsson et al. [10], effluent from a pharmaceutical wastewater treatment plant (WWTP) contained approximately 30 to 33 mg/L of total FQs. FQs can accumulate in the soil, bottom sediments, and water reservoirs [1,2,4,11,12,13,14].

One FQ, namely, enrofloxacin (ENR), was detected in chicken manure at a maximum concentration exceeding 1420 mg/kg [15]. Very high concentrations of these antimicrobials have also been found in the water and sediments of shrimp aquaculture. Another FQ, norfloxacin (NOR), was detected in sediments at a concentration of up to 2615 mg/kg based on the weight of the wet mud [16].

Due to their stable chemical structure, FQs are highly resistant to biodegradation in the natural environment [2,14,17,18]. They are only partially degraded in activated sludge (AS); therefore, they cannot be effectively removed in conventional biological/mechanical WWTPs [14,19]. Consequently, high concentrations of FQs have been frequently detected in the WWTP effluent, and significant amounts remain undecomposed in AS. Based on literature data, the content of FQs in this matrix can range from 0.1 to even 98,000 µg/kg [14]. Such high concentrations of antibiotics are expected to have a negative impact on the local microbiocenosis structure [13]. In the environment, FQs occur most often at sub-inhibitory concentrations, in nanogrammes per litre. However, as reported by Andersson et al. and Paul et al. [20,21], antibiotics, even at such low concentrations, can also promote the development of resistant microorganisms.

Some studies have confirmed that FQs are partially decomposed by sunlight [14,18,22,23,24,25,26], although they are much more easily degraded by the photocatalytic process. These reactions take place under UV or VIS irradiation in the presence of TiO_2_, ZnO, ZrO_2_, WO_3_, g-C_3_N_4_, and CoAl-LDH as a photocatalyst and have been described in detail in the literature [21,23,27,28,29,30,31,32,33]. Unfortunately, the application of photocatalysis for the mineralisation of micropollutants in wastewater faces many obstacles. This is a non-selective process, and the complete mineralisation of antimicrobials requires a long irradiation time and is very expensive [27,29,34]. After a much shorter exposure time, micropollutants such as pharmaceuticals may undergo partial decomposition, leaving organic degradation products (ODPs) in the post-reaction mixture [27,28,29,32,35]. These compounds can still possess antimicrobial activity and be toxic to higher organisms. After their introduction into the environment, they can be as hazardous as the parent FQs.

One of the proposals for using photocatalysis for the treatment of wastewater containing antibiotics is to combine it with subsequent biodegradation [35,36]. This solution allows for the reduction of the total operating cost of wastewater treatment and the simultaneous removal of ODPs. However, these products must be biodegradable and non- toxic to microorganisms. The ecotoxicity of FQ degradation products formed during advanced oxidation processes has been reported in the literature [24,25,26,33,37,38,39]. The majority of researchers have confirmed that these products were more ecotoxic or genotoxic than the parent FQs [24,25,26,37,38,39]. However, only a single type of biotest was often used to assess ecotoxicity in the aforementioned studies.

The aim of our study was to identify organic intermediates formed as a result of photocatalytic degradation of NOR and ENR and to assess their residual antimicrobial activity. The study also included the determination of the in vitro antimicrobial activity of solutions resulting from the incomplete degradation of FQs towards the selected bacterial strains. Furthermore, we investigated the stability of identified ODPs in sterile deionised water, natural surface waters, and in the presence of activated sludge from a WWTP. Finally, we performed an in silico assessment of the ecotoxicity of these intermediates towards algae, daphnids, and fish.

## 2. Results and Discussion

### 2.1. Kinetics of Photocatalytic Degradation

**Figure 2:**
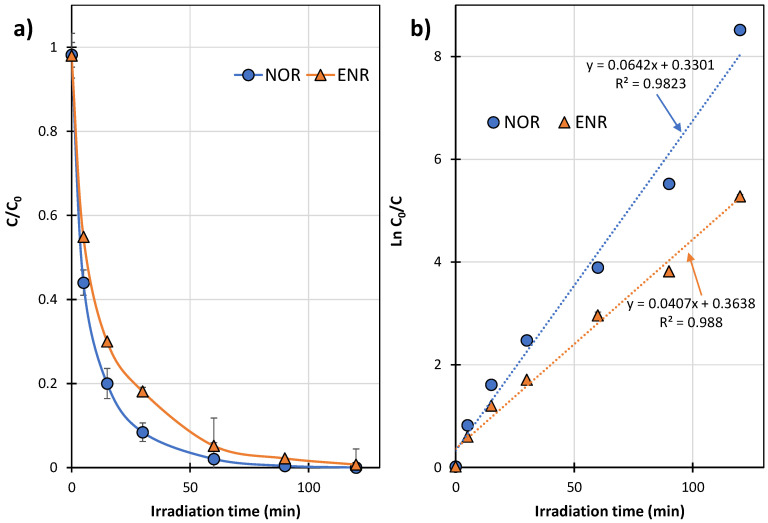
Dynamics of the photocatalytic degradation of NOR and ENR in solutions irradiated in the presence of TiO_2_-P25: (**a**) changes in FQ concentration, (**b**) a plot of ln(C_0_/C) vs. time.

**Figure 3:**
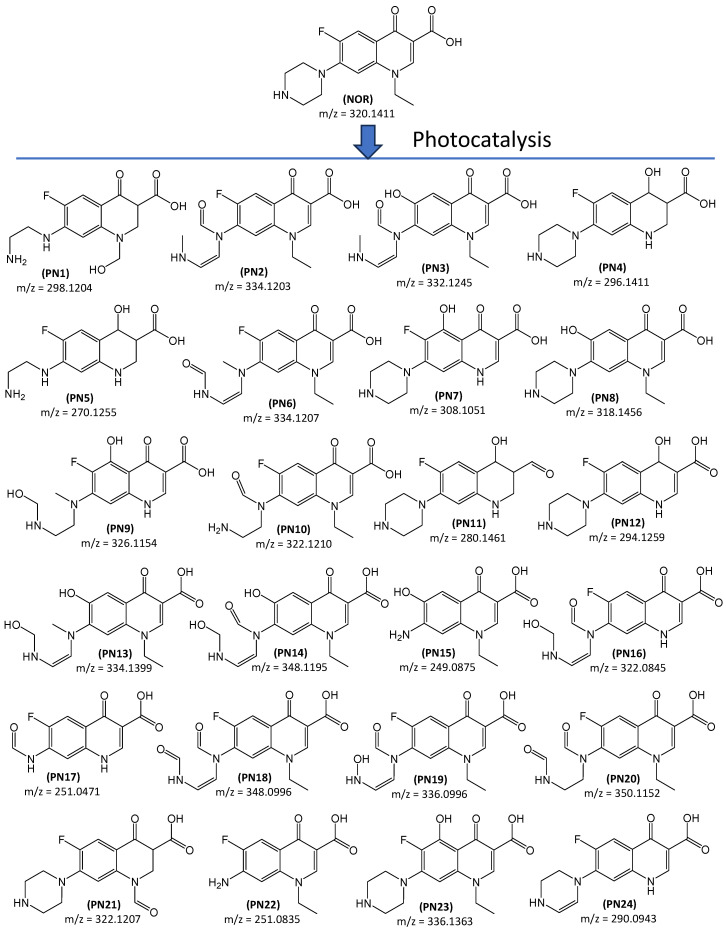
Proposed degradation products of NOR. The order of ODPs is related to their retention times (Table S3, SM).

**Figure 4:**
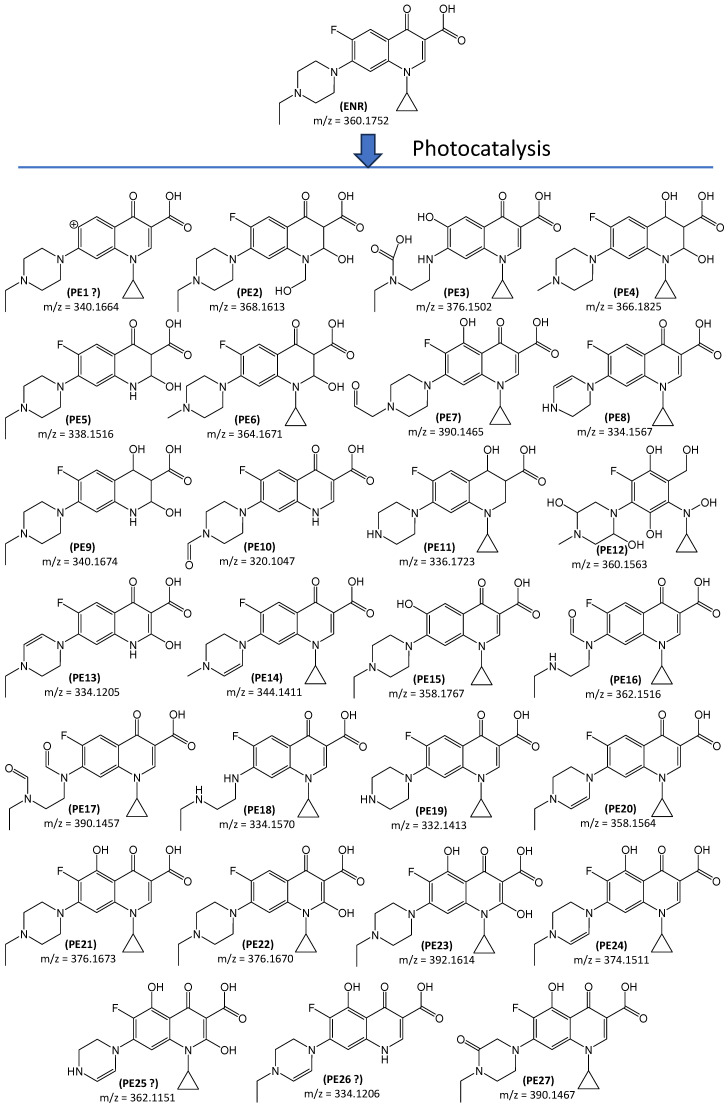
Proposed degradation products of ENR. The order of ODPs is related to their retention times (Table S4, SM). The formulas for compounds PE1, PE25, and PE26 were proposed based on a single fragment ion.

The dynamics of photocatalytic degradation of NOR and ENR in the presence of TiO_2_-P25 are shown in Figure 2a. The high correlation (R^2^) for the linear relationship of *ln C_0_*/*C* vs. time (Figure 2b) confirms that the degradation of both antibiotics follows a pseudo-first-order process. This is consistent with the kinetic model of photocatalytic degradation of FQs presented by other researchers [29,32,35]. The degradation rate constant (k) values of NOR and ENR determined in the experiment were 0.0642 ± 0.0039 and 0.0407 ± 0.0020 min^−1^, respectively. The structure of ENR differs from that of NOR due to the presence of an ethyl group at the nitrogen atom of piperazine and a cyclopropyl group at the nitrogen atom of the quinolone moiety (Figure 3 and Figure 4). Most likely, one of these groups could hinder degradation of ENR. However, the degree of degradation of both antibiotics was >99% after 120 min of UVA irradiation.

### 2.2. Photocatalytic Degradation Products

In NOR and ENR solutions irradiated for 120 min with TiO_2_-P25, the total organic carbon (TOC) content decreased only slightly, by approximately 20 and 11%, respectively. This indicates that both antibiotics transformed into other organic compounds. Figure 3 and Figure 4 present the predicted molecular formulas for the degradation products of NOR and ENR. Detailed data for identified products are included in Supplementary Material (Tables S3 and S4, SM). For data interpretation, monoisotopic peaks of ODPs with signal-to-noise ratios equal to or greater than 3 were extracted from the total ion current (TIC) chromatograms. In total, twenty-four ODPs of NOR and twenty-seven ODPs of ENR are summarised in Figure 3 and Figure 4.

Hydroxyl radicals and/or superoxide radicals are of key importance in the degradation processes of FQs carried out under UV or VIS irradiation and in the presence of various catalysts [21,27,28,35,40,41]. They can participate in oxidative dehydrogenation and decyclisation processes, dealkylation, and hydroxylation. Zhang et al. [27] have reported that oxidative degradation of ENR typically begins with the attack of the radicals on the piperazine ring. As a result, dehydrogenation and subsequently the piperazine ring-opening and/or carbon atom oxidation occur. The structures of the majority of ODPs, i.e., PN1-PN3, PN5, PN6, PN9, PN10, PN13-PN20, PN22, and PN24, as well as PE3, PE8, E14, PE16-PE18, PE20, and PE27, are in agreement with the mechanism presented above. Some ODPs can form as a result of the oxidation of substituents on the nitrogen atom. Eleven of them were formed during the NOR degradation (PN1, PN4, PN5, PN7, PN9, PN11, PN12, PN16, PN17, PN21, and PN24), and fifteen of them as a result of ENR degradation (PE2, PE4, PE5-PE7, PE8-PE14, PE19, PE25, and PE26). In the ENR molecule, the cyclopropyl group exhibited a low susceptibility to oxidation. We proposed only one product (PE2) formed by direct oxidation and five products (PE5, PE9, PE10, PE13, and PE26) in which the cyclopropyl group was eliminated.

The bicyclic quinolone structure in FQs is probably also resistant to degradation, as it occurs unchanged in most of the identified ODPs. We identified only one transformation product of ENR in which the heterocyclic pyridine ring was degraded (PE12). However, it cannot be excluded that more such products may be formed during oxidation of FQs. They are probably unstable and undergo further rapid rearrangement and decomposition under the experimental conditions. Therefore, the potential impact of such ODPs on the environment will be marginal. Products of hydrogenation/reduction of the heterocyclic ring and/or the carbonyl group at C-4 were also identified in solutions after degradation of NOR (PN1, PN4, PN5, PN11, PN21) and ENR (PE2, PE4-PE6, PE9, PE11). The probable mechanism of reduction of these groups during photocatalysis has been described by Valenzuela et al. [42]. In addition, we found products resulting from the elimination or substitution of the fluorine atom in the FQ molecule. In NOR solutions, these were ODPs marked with symbols PN3, PN8, PN13-PN15, while in ENR solutions, these were compounds PE1, PE3, and PE15. Similar degradation products have also been described in the literature [27,28,29].

The hydroxylation of the aromatic ring of the bicyclic quinolone structure during photodegradation of cyclic organic compounds has been reported in the literature [28,43]. We found several ODPs which formed as a result of this mechanism, three after NOR photocatalysis (PN7, PN9 and PN23) and seven after ENR transformation (PE7, PE21, PE23-PE27). Almost all identified ODPs had a preserved 4-pyridone-3-carboxylic acid moiety. Since this group acts as a pharmacophore, such ODPs are expected to exhibit antibacterial activity [44]. This phenomenon is unfavourable because, after entering the environment, these compounds can have a negative impact on local biocenosis.

**Figure 5:**
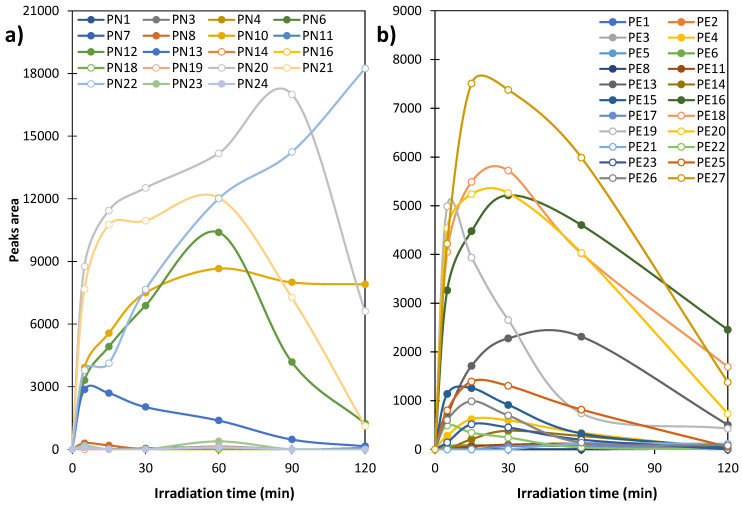
Changes in the intensity of peak area of ODPs during UVA irradiation of (**a**) NOR and (**b**) ENR solutions.

The dynamics of changes in ODP concentration during the UVA irradiation of FQ solutions in the presence of TiO_2_ P25 are shown in Figure 5. The figure does not include the products whose relative intensity was significantly low in all samples.

In NOR solutions after UVA irradiation, large peak areas corresponding to ODPs were observed only for compounds marked as PN10, PN12, PN13, PN20, PN21, and PN22 (Figure 5a). During irradiation, the peak areas of products PN12, PN13, PN20, and PN21 initially increase and then decrease, suggesting that they undergo further transformations. In turn, the concentration of PN10 stabilises and the concentration of PN22 increases systematically with prolonged irradiation time. In compound PN22 the heterocyclic ring is reduced to an amino group (Figure 3), indicating that it may be a product of subsequent transformations of many other identified ODPs [27].

In ENR solutions, the maximum concentration of identified ODPs occurred between 5 and approximately 50 min of UVA irradiation (Figure 5b). Longer irradiation resulted in their further decomposition. We did not identify any ODPs whose concentrations increased systematically throughout the experiment. Unfortunately, high peaks corresponding to potentially biologically active substances (with preserved pharmacophore) were identified in the chromatograms even after 120 min of UVA irradiation of NOR and ENR solutions.

### 2.3. Fate of NOR, ENR, and ODPs in the Selected Aquatic Ecosystems

Reagent concentrations were set based on previously established microbial toxic concentration (MTC) values (Section 2.4). These levels were sufficiently high to observe changes in growth inhibition of the test microorganisms and to identify the ODPs. Simultaneously, the FQ concentrations after photocatalysis remained low enough to prevent the inactivation of microorganisms in activated sludge.

As already mentioned, the photocatalytic process is an effective method to decompose FQs in the aquatic environment. However, depending on the process conditions, complete photocatalytic mineralisation of both FQs and their intermediates would require two to four times longer irradiation time [28,31,45]. For economic reasons, only partially photocatalytically treated wastewater could be released into the environment. As shown in Section 2.2, most identified ODPs have a preserved pharmacophore and, therefore, can potentially impact the microbiocenosis [44]. For this reason, it is necessary to know their further fate in the environment.

To achieve this goal, an experiment was carried out in which FQ solutions after 60 min of irradiation-containing ODPs (Figure 5) and residues of NOR (4.0 ± 1.1 × 10^−3^ mmol/L) or ENR (1.1 ± 0.2 × 10^−2^ mmol/L) were used. After catalyst removal, the solutions were mixed in a 1:1 ratio with deionised water, AS from WWTP, river water, lake water, or water from the source. The characteristics of the environmental samples are presented in Table S1, SM. Deionised water and the source water were sterile. The experiment was carried out under aerobic conditions for 28 days (the samples with deionised water were not aerated).

**Figure 6:**
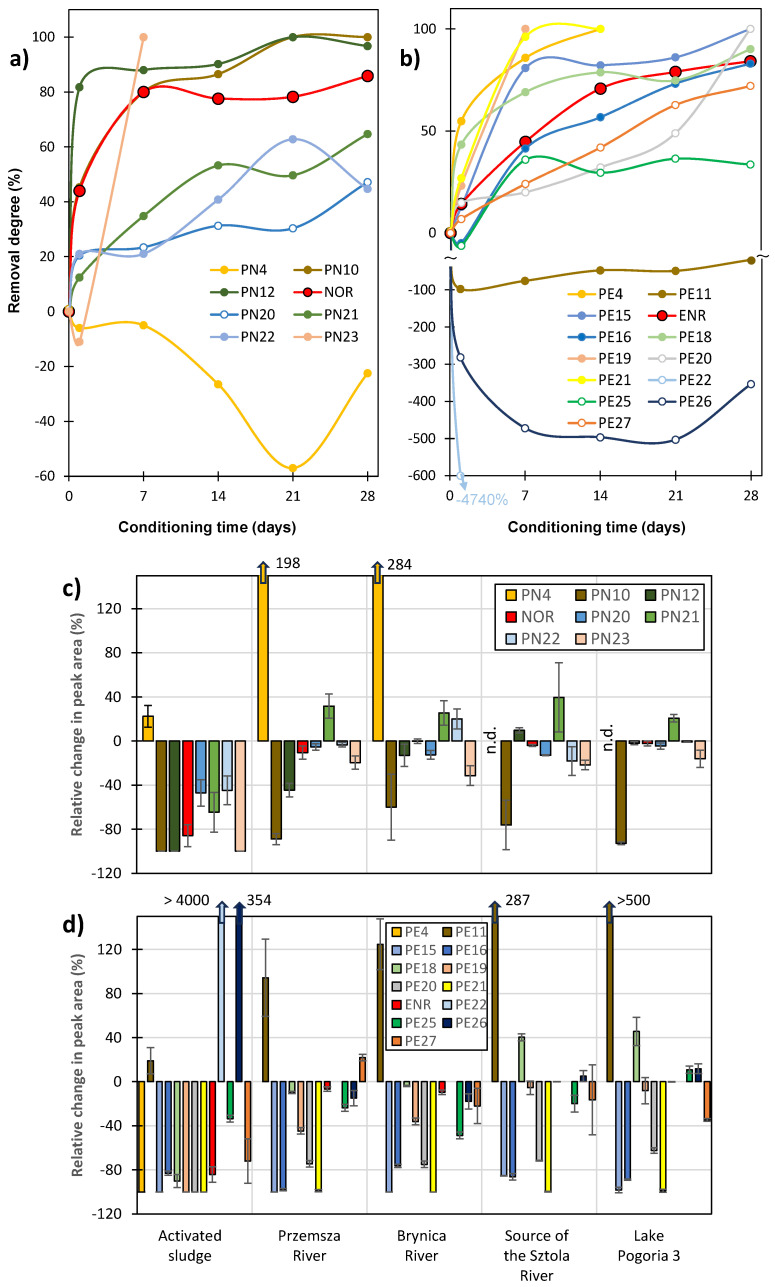
Degree of removal/degradation of (**a**) NOR and (**b**) ENR and their ODPs during conditioning with AS under aerobic conditions. Relative changes in the intensity of (**c**) NOR and (**d**) ENR peaks (marked in red) and their ODPs after 28 days of conditioning under aerobic conditions.

The composition of FQ solutions in deionised water and under sterile conditions changed only slightly over 28 days (Figures S4 and S5, SM). Significant changes in the intensity of only a few peaks corresponding to PN10, PE4, PE23, and PE24 were observed. The experiment demonstrated that the concentrations of both studied FQs and most of their ODPs were almost unchanged, and they were stable without aeration, natural sunlight, or biological material in the samples. Relative changes in the concentrations of NOR and ENR and their ODPs during conditioning with AS under aerobic conditions are shown in Figure 6.

After 28 days of conditioning, the concentrations of NOR and ENR in AS decreased by 86 ± 10% and 84 ± 7%, respectively. Many of their ODPs were almost completely decomposed. On the other hand, the degradation of some NOR products (PN20 and PN22) and the ENR product (PE25) did not exceed 50%, which means that these substances would not be effectively removed in biological WWTPs. The concentrations of only one ODP of NOR (PN4) and three of ENR (PE11, PE22, and PE26) increased during the experiment in AS. The degree of removal of these ODPs is shown by negative values in Figure 6. These compounds were possibly persistent products of aerobic biodegradation and could be formed by hydrogenation or hydroxylation of other ODPs.

The summary of relative changes in the peak areas of FQs and their ODPs after 28 days of conditioning under aerobic conditions is shown in Figure 6c,d. We took into account only those products for which the peak area was sufficient to assess the dynamics of biodegradation in the highly complex matrix of AS.

The concentration of NOR in environmental samples practically did not change during 28 days of conditioning. Only one product of NOR (PN10) was further degraded under these conditions (Figure 6c). This compound also decomposed in the source water, i.e., under abiotic conditions. Under the same conditions, the concentrations of two ODPs of NOR (PN12 and PN21) increased. In turn, in the water from the Przemsza and Brynica rivers, a significant increase in PN4 concentration was observed and, to a lesser extent, an increase in PN21 concentration. Similar effects occurred in solutions containing ENR and its ODPs (Figure 6d). In all the natural ecosystems studied, the concentrations of PE15, PE16, PE20, and PE21 decreased by more than 50%. Other ODPs and ENR were decomposed to a much lower extent. Compounds PE11 and PE18 were probably products of further abiotic transformations of ODPs. In turn, compounds PE25-PE27 could be both products and substrates in processes that occur at a similar rate. These results indicate that both FQs and some ODPs are stable and can impact the ecological balance of the microbiosphere for a long time.

### 2.4. Changes in the Toxicity of Solutions to Test Microorganisms (MARA^®^)

**Figure 7:**
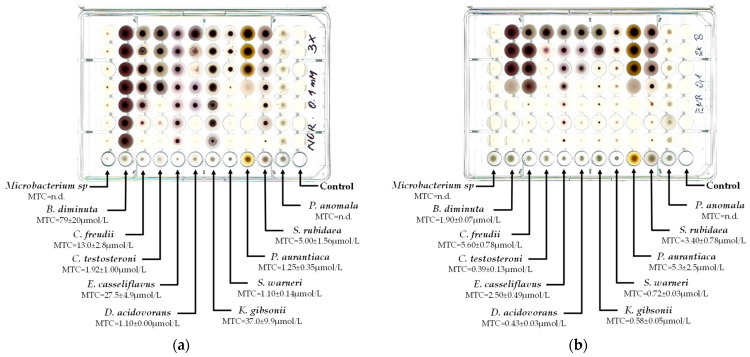
MARA^®^ plate image with gradient of (**a**) NOR and (**b**) ENR concentration and MTC values determined for the test strains.

To assess the effect of the FQs studied and their ODPs on real microbiocenosis, the commercial MARA^®^ test was used. This is a bioassay for chronic toxicity testing based on MTC values, using ten bacteria of different taxonomies and yeasts as bioindicators [46,47,48,49]. Details of the bioassay experiments and procedures used are described in SM. Figure 6 shows MARA^®^ plate images with a concentration gradient of NOR and ENR solutions (from 4.1 × 10^−4^ to 1.0 × 10^−1^ mmol/L), and the MTC values determined for the test microorganisms. These values are inversely proportional to the sensitivity of microbes to potentially toxic substances [50]. Furthermore, the MTC value is close to the lowest observed effect concentration (LOEC), which is the lowest concentration that allows the observation of statistically significant growth inhibition [41]. Further interpretation of results does not include *Microbacterium* sp. and *P. anomala* (columns 1 and 11, Figure 7). The slight growth of these strains did not allow for the correct determination of MTC values.

In the range of concentrations used, ENR inhibited the growth of all test microorganisms and was approximately 10 times more toxic than NOR. The mean MTC values for NOR and ENR were 18.5 and 2.3 μmol/L, respectively. However, for individual strains, toxic effects were observed at lower concentrations of NOR and ENR (1.10 ± 0.14 and 0.39 ± 0.13 μmol/L, respectively). Both FQs inhibited the growth of *C. testosteroni*, *D. acidovorans*, *K. gibsonii*, and *S. warneri* to the greatest extent.

**Figure 8:**
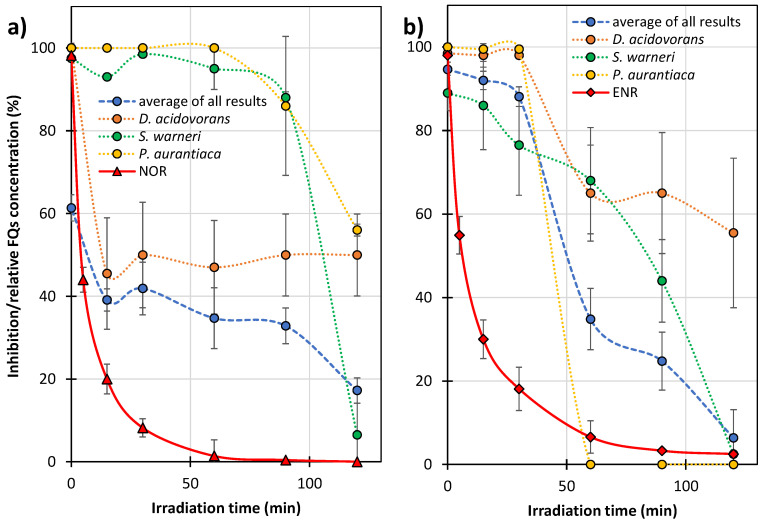
Effect of the irradiation time on the relative concentration of FQs (solid red line) and growth inhibition (dotted lines) of the test bacteria (MARA^®^ bioassay) in solutions containing ODPs and residues of NOR (**a**) or ENR (**b**). Blue dashed line—average of all results for nine microbial strains (explanation in the text).

Figure 8 shows the changes in growth inhibition of microorganisms (MARA^®^) in the presence of the FQ solutions after UVA irradiation with TiO_2_, and the relative changes in NOR and ENR concentrations. For clarity, only the changes in the mean growth inhibition of nine microorganism strains and the growth inhibition of the most sensitive microorganisms tested, i.e., *D. acidovorans*, *S. warneri*, and *P. aurantiaca*, are presented in the Figure.

These microbes were considered representative in order to observe changes in the toxicity of FQ samples. With extended irradiation time, the toxicity of the FQ solutions decreased. However, this effect was much slower than that of the FQ degradation. This confirms our previous assumption that many of the formed ODPs contained a preserved pharmacophore responsible for bactericidal properties (Section 2.2).

The post-degradation solution of ENR resulted in less growth inhibition of the most sensitive strains of the MARA^®^ test. This indicates that it was less toxic than the analogous NOR solution, despite the fact that ENR was more toxic and degraded more slowly (Figure 2 and Figure 8b). The ENR solution showed low toxicity after 120 min photocatalysis and, in its presence, the mean growth inhibition of the nine strains of bacteria was <10% (Figure 8b). However, the solutions of both FQs after the end of UV irradiation still showed high toxicity to *D. acidovorans* (growth inhibition at the 50% level). Furthermore, the solution after NOR degradation was also toxic to *P. aurantiaca* (Figure 8a). This indicates that, under the conditions used, the FQ solutions after photocatalysis can result in disturbances in environmental microbial communities.

**Figure 9:**
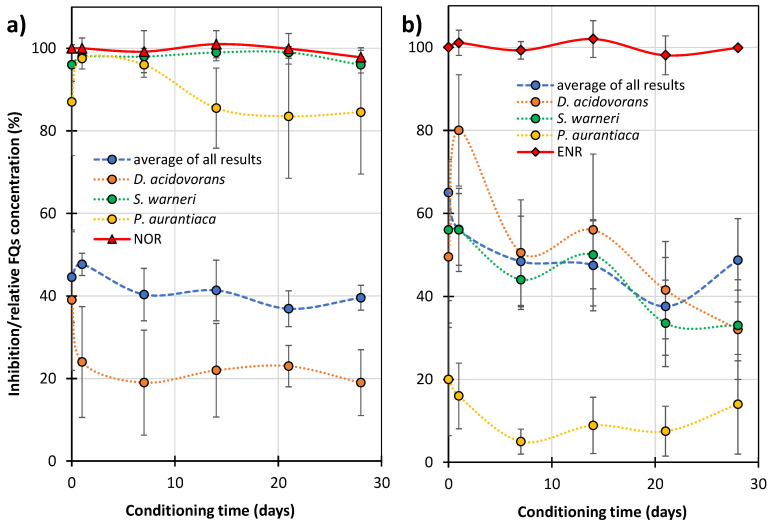
Effect of the conditioning time of Lake Pogoria 3 water with samples after photocatalysis on the relative FQs concentration (solid red line), and inhibition (dotted lines) of bacterial growth (MARA^®^ test) in solutions containing ODPs and residues of NOR (**a**) and ENR (**b**). Blue dashed line—average of all results for nine microorganisms’ strains (explanation in the text).

Figure 9 shows the growth inhibition of microorganisms from Lake Pogoria 3 by solutions containing residues of FQs and ODPs. Since the lake water was highly pure (Table S1, SM), we assumed that this would not affect the results of inhibition of microbial growth by FQs studied and their ODPs.

After 28 days of conditioning Lake Pogoria 3 water with solutions containing NOR residues and its ODPs, only a slight decrease (from 39 ± 17% to 19 ± 8%) in the growth inhibition of *D. acidovorans* was observed (Figure 9a, orange dotted line). The mean growth inhibition of nine strains practically did not change (blue dashed line). Solutions containing ENR and its ODPs caused a decrease in the average growth inhibition from 65 to 49%, (Figure 9b, blue dashed line) and a decrease in the growth inhibition of *S. warneri* from 56 to 33% (green dotted line). This result is consistent with the degradation results (Figure 6) and confirms that ODPs of ENR are more susceptible to biodegradation in the environment than those of NOR.

### 2.5. In Silico Prediction of Products Toxicity

**Figure 10:**
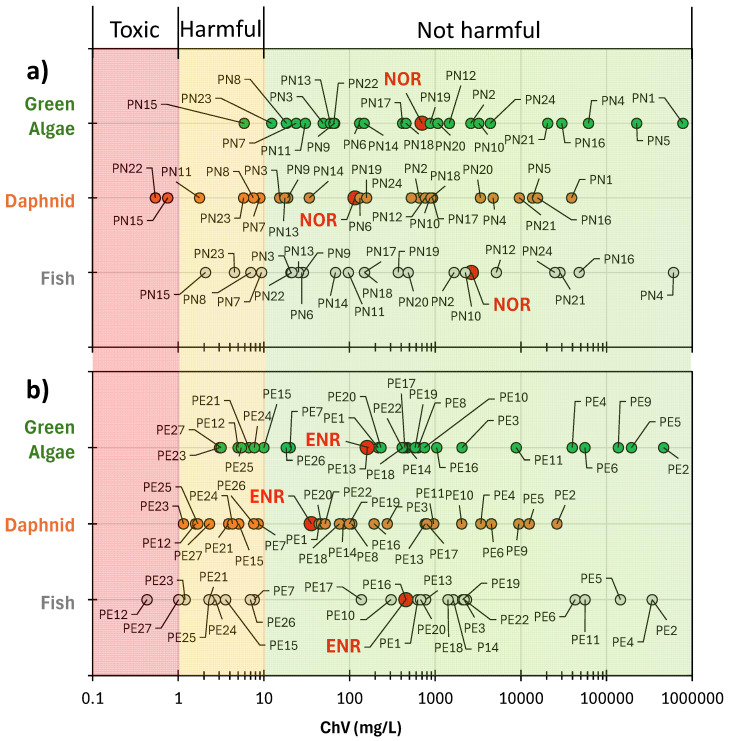
Chronic toxicity values (ChV) of FQs and their ODPs predicted by ECOSAR; (**a**) NOR, (**b**) ENR.

The predicted chronic toxicity values of FQs and their identified ODPs to aquatic organisms were determined using the in silico approach. ECOSAR 2.2 software is a widely recognised tool for predicting acute and chronic toxicity based on the quantitative structure-activity/ecotoxicity relationship [51]. The resulting values are shown in Figure 10.

It was found that both FQs and most of their ODPs were classified as non-ecotoxic substances to organisms from the aquatic environment, i.e., green algae, daphnids, and freshwater fish. Two NOR degradation products were classified as toxic to daphnids (PN15 and PN22; Figure 10a), and four compounds (PN7, PN8, PN11, and PN23) were considered harmful. Intermediates PN7, PN8, PN15, and PN23 were regarded as harmful to fish and PN15 as harmful to algae. The peak corresponding to PN22 (1-ethyl-6-fluoro-7-amino-4-oxo-1,4-dihydroquinoline-3-carboxylic acid) had a high intensity, suggesting that this toxic compound was present in substantial concentrations in the post-reaction solution. Furthermore, its amount increased with prolonged irradiation time and with an increasing degree of NOR degradation (Figure 5a). Biodegradation in AS proved to be only a partially effective method to remove this compound (Figure 6a).

Two ODPs of ENR, i.e., PE12 and PE27, were considered potentially toxic to fish, while PE15, PE21, and PE23-PE26 were considered potentially harmful to all organisms tested (Figure 9b). Additionally, PE7 was potentially harmful to fish and daphnids, and PE12 to daphnids and algae. The high intensity of the peak corresponding to PE27 (1-cyclopropyl-5-hydroxy-6-fluoro-7-(4-ethyl-3-oxopiperazin-1-yl)-4-oxo-1,4-dihydroquinoline-3-carboxylic acid) may indicate that this compound was present in significant amounts in the solutions after irradiation (Figure 5b). Furthermore, the magnitude and type of changes in the PE27 peak intensity in the conditioned solutions indicate that its biodegradation in natural ecosystems is not complete (Figure 6d). This suggests that wastewater insufficiently treated by the photocatalytic method, still containing PE27, may also pose a threat to aquatic vertebrates after introduction into the aquatic environment. On the other hand, this compound was partially biodegraded in AS (72 ± 20%) after 28 days of conditioning (Figure 6b). Nine ODPs identified after ENR degradation (PE7, PE12, PE15, PE21, and PE23-PE27) were considered hazardous (Figure 10b). As a result of biodegradation in AS, the intensity of the peak corresponding to PE26 increased by more than 350% (Figure 6d).

The results obtained using the in silico tool are not fully consistent with the experimental data reported in the literature. In some of them, FQs have been shown to inhibit the growth of cyanobacteria, green algae, and duckweed [22]. This may indicate that solutions containing complex degradation products of FQs, when introduced into the environment, could be even more hazardous to the aquatic ecosystems than these predictions suggest. However, we observed a significant decrease in antibacterial activity of solutions containing FQs residues after the photocatalytic degradation. This indicates a limitation of the biological risk of antibiotic resistance in the environment, which is the result of incomplete degradation of antibiotics by traditional wastewater treatment.

## 3. Materials and Methods

### 3.1. Reagents

Two antimicrobials, namely, NOR and ENR (both >99% purity), were purchased from the manufacturer (Sigma-Aldrich, St. Louis, MO, USA). Commercial Aeroxide^®^ TiO_2_-P25 (Evonik, Essen, Germany) was used as a photocatalyst (Supplementary Materials, SM). Phytone peptone (Becton, Dickinson & Co., Franklin Lakes, NJ, USA) and 2,3,5-triphenyltetrazolium chloride (TZR, purity p.a., POCH, Gliwice, Poland) were used in microbiological tests. Furthermore, NaOH (purity p.a., Chempur, Piekary Śląskie, Poland), water for LC-MS (Chromasolv^®^; Fluka-Analytical, Buchs, Switzerland), acetonitrile for LC-MS (LiChrosolv^®^; Supelco, Bellefonte, PA, USA), formic acid (98–100% for LC-MS, LiChropur^®^; Supelco, Bellefonte, PA, USA) and leucine enkephalin (Waters, Milford, MA, USA) were used in experiments.

### 3.2. Photocatalytic Process

The FQ concentrations used in experiments were similar to those found in wastewater from the pharmaceutical industry [22]. Their solutions (0.2 mmol/L) were individually prepared in deionised water (conductivity, φ ~ 0.6 mS/cm). A quantity of 100 mL of each solution and 50 mg of solid TiO_2_-P25 were added to crystallisers (diameter of 11 cm, capacity of 500 mL). The amount of photocatalyst was optimised in our previous studies with respect to the photocatalytic degradation of other antibiotics [52,53,54]. The pH of suspensions was adjusted to 6.5 ± 0.5 using NaOH solution (1 mmol/L) due to their intended use in biodegradation experiments. To establish an adsorption–desorption equilibrium, the suspensions were stirred for 15 min in the dark and then irradiated with UVA radiation at an intensity of 13.6 W/m^2^ (ACTYNIC BL TL 40 W/10 lamps, λ_max_ = 366 nm, Philips, Amsterdam, Netherlands). The irradiation intensity was controlled by a quantum-photo radiometer (DO972, Delta OHM, Caselle Di Selvazzano, Italy). The setup for photocatalytic degradation is shown in Figure S1, SM. During irradiation, the solutions were kept in free contact with air, and their temperature was maintained in the range of 293–297 K. Samples were taken from irradiated solutions at fixed time intervals, immediately filtered (nylon syringe filter, 25 mm/0.45 µm, Labfil, Hangzhou, Zhejiang, China), and analysed by UPLC-PDA/QTOF.

### 3.3. Sample Conditioning (Biodegradation)

FQ solutions designed for biodegradation experiments were irradiated in the presence of TiO_2_-P25 for 60 min (Section 3.2). The irradiation time was determined based on the previously determined kinetics of photocatalytic degradation of FQs (Section 2.1). The catalyst was removed from the suspension by filtration (sterile syringe filter, 28 mm/0.2 µm, Minisart^®^ NML Plus, Sartorius, Getynga, Germany). Sterile solutions containing undegraded antibiotics and their ODPs were mixed with homogenised AS, with environmental samples from the Przemsza River, the Brynica River, the source of the Sztoła River and Lake Pogoria 3, or with deionised water (Table S1, SM) in a ratio of 1:1 (V/V). AS was collected from the aerobic and denitrification chamber of the WWTP in Sosnowiec (Poland). The mixed liquor suspended solids concentration of AS was 3.1 ± 0.5 g/L. The mixtures were transferred to 50 mL cylinders, then thermostated (284 ± 0.2 K) and aerated with sterile air for 28 days. Air flow in the cylinders was 2.5 ± 0.5 mL/min. The samples were protected from direct sunlight. Reference solutions (without FQs) were subjected to an analogous procedure. Additionally, samples of FQs with deionised water were placed in a thermostat in closed vessels; these samples were treated as controls. Figure S2 (SM) depicts the schematic view of the experimental setup during conditioning (biodegradation) under aerobic conditions. Aliquots from the cylinders were withdrawn after 0, 1, 7, 14, 21, and 28 days of conditioning, immediately filtered (nylon syringe filter 25 mm/0.45 µm, Labfil, Zhejiang, China), and analysed by UPLC-PDA/QTOF.

### 3.4. UPLC Analysis

Chromatographic analysis was performed using an ultra-performance liquid chromatography unit (Acquity UPLC I Class System, Waters Corp., Milford, MA, USA) with an Acquity UPLC BEH C18 column (2.1 mm × 100 mm, 130 Å, 1.7 µm) and two detectors (PDA and QTof, Xevo G2-XS, Waters Corp., Milford, MA, USA). The mobile phase was composed of solvents A (H_2_O with 0.01% HCOOH) and B (CH_3_CN with 0.01% HCOOH) with gradient elution as follows: 0 min at 95% A, 3.5 min at 80% A, 6.5 min at 70%, A 7.5 min at 70% A, and 8.3 min at 95% A. The flow rate was 0.350 mL/min, the column temperature was 308 K, and the injection volumes were 1 µL and 5 µL. The concentration of FQs in effluent was monitored using a PDA detector at a wavelength (λ) of 278 nm. The ODPs of NOR and ENR were identified by comparing the chromatograms of samples after degradation with reference solutions (without FQs). The exact monoisotopic masses of protonated molecules ([M + H]^+^) of ODPs, and their fragment ions obtained at the collision energy between 10 and 35 V were recorded on the QTof (ESI+) detector. MassLynx^®^ (V4.1, Waters, Milford, MA, USA) software was used for the analysis of the elemental composition of ODPs. Their structural formulas were created using the ChemDraw^®^ Std (ver. 25.0.2.14) with the Analysis Windows package (Cambridge Soft, Cambridge, MA, USA). Small-molecule aliphatic products were not identified. The ionisation conditions and operating parameters for the Xevo Qtof detector are given in Table S2 (SM).

### 3.5. Analysis of Microbial Ecotoxicity (MARA^®^ Bioassay)

The chronic toxicity of solutions containing antibiotics and their ODPs was evaluated using the MARA^®^ bioassay (NCIMB Ltd., Aberdeen, Scotland, UK). Experiments were conducted using procedures described by Adamek et al. [52] and Wadhia [46,47] and their detailed data are given in SM.

### 3.6. Analysis of Total Organic Carbon

Total organic carbon (TOC) analysis was performed using the LCK385 cuvette test (HACH LANGE, Loveland, CO, USA). The tests were carried out according to the manufacturer’s procedure [55]. The results were read from a DR 3900 spectrophotometer (HACH LANGE, Loveland, CO, USA).

### 3.7. Turbidity Determination

The turbidity of samples was measured with a spectrophotometer DR 3900 using the programmed method No. 747.

### 3.8. Electrochemical Measurements

The pH and specific conductivity (φ) of samples were measured using a laboratory multimeter HQ440D (HACH LANGE, Loveland, CO, USA).

### 3.9. Microorganisms Counts

The counts of microorganisms in environmental samples were determined using the Heterotrophic Plate Count Compact Dry™ AQ Test (Shimadzu Diagnostics Europe, Paris, France). Tests were performed following the manufacturer’s procedure [56].

### 3.10. In Silico Prediction of Toxicity

The potential toxicity of FQs and their ODPs to aquatic organisms was determined using the Ecological Structure–Activity Relationship Model (ECOSAR, ver. 2.2, US EPA). The ECOSAR can be used to predict, among others, the chronic toxicity of chemicals to fish, daphnids, and green algae. Details about the application are available on the United States Environmental Protection Agency (US EPA) website [51].

## 4. Conclusions

FQs undergo photocatalytic degradation in the presence of TiO_2_ according to pseudo-first-order kinetics. Nitrogen atom substituents in the FQ molecules can hinder their photocatalytic mineralisation. After degradation, the structural formulas of 24 and 27 NOR and ENR intermediates, respectively, were established. Most of the complex organic products of photocatalytic degradation of FQs have a preserved pharmacophore and can exhibit antibacterial activity. After 120 min of irradiation, the average inhibition of microorganisms in the MARA^®^ bioassay caused by NOR and ENR solutions decreased from 61 to 17% and from 95 to 6%, respectively. Complete elimination of this activity is possible, but it requires a much longer irradiation time than needed for the decomposition of the parent FQs.

In the natural environment, the FQs studied, and some ODPs, are stable and can affect the microbiosphere for a long time. In Lake Pogoria 3 water, after 28 days of conditioning, the inhibition of the MARA^®^ microorganisms caused by solutions containing NOR and ENR residues, and ODPs decreased only from 44% to 40% and from 65% to 48%, respectively. In addition, some of the identified ODPs can also be toxic to aquatic organisms other than bacteria. Based on the ECOSAR software, it was determined that six and nine ODPs of NOR and ENR, respectively, were toxic and/or hazardous to aquatic organisms. FQs remaining after photocatalysis and most ODPs can be removed from wastewater during aerobic biodegradation using AS. The combination of these two processes significantly reduces the ecotoxicity of solutions containing FQs residues. Therefore, it cannot be ruled out that this will limit the generation of ARGs in the environment. However, one of the toxic ODPs is resistant to biodegradation under the conditions used. This indicates that the substance will not be effectively removed in biological WWTPs and can still cause disturbances in environmental microbiocenoses.

## Data Availability

The original contributions presented in this study are included in the article/Supplementary Material. Further inquiries can be directed to the corresponding author.

## Supplementary Materials

The following supporting information can be downloaded at: https://www.mdpi.com/article/10.3390/ijms27052099/s1.

## Abbreviations

Table following abbreviations are used in this manuscript:

FQ	Fluoroquinolone
WWTP	Wastewater Treatment Plant
NOR	Norfloxacin
ENR	Enrofloxacin
AS	Activated Sludge
ODP	Organic Degradation Product
TOC	Total Organic Carbon
TIC	Total Ion Current
MTC	Microbial Toxic Concentration
LOEC	Lowest Observed Effect Concentration
TZR	2,3,5-Triphenyltetrazolium Chloride
p.a.	Pure Pro Analysis
LC-MS	Liquid Chromatography–Mass Spectrometry
UPLC	Ultra-Performance Liquid Chromatography
PDA	Photodiode Array Detector
QTOF	Quadrupole Time-of-Flight
ESI+	Electrospray Ionisation in positive mode
US EPA	United States Environmental Protection Agency
