# Supplementary material for: Environmental Impact of Fluoroquinolones and Their Photocatalytic Transformation Products: Degradation with Activated Sludge and in Surface Waters, Change in Antimicrobial Activity and Ecotoxicity
Source: Int J Mol Sci. 2026 Feb 24;27(5):2099. doi: 10.3390/ijms27052099 (PMC12984707; doi:10.3390/ijms27052099)
Supplement: Supplementary file 1 [file ijms-27-02099-s001.zip › ijms-4152846-supplementary.pdf]

Supplementary Materials

Environmental impact of fluoroquinolones and their photocatalytic transformation products: degradation with activated sludge and in surface waters, change in antimicrobial activity and ecotoxicity.

Technical data of Aeroxide® TiO_2-P25 (Evonik) available on https://products.evonik.com/assets/or/ld/AEROXIDE_TiO2_P_25_TDS_EN_EN_TDS_PV_52043891_en_GB_WORLD.pdf

- appearance white solid
- delivery form free-flowing powder
- loss on drying < 1.5%
- pH-value 3.5 - 4.5
- SiO_2 content < 0.2%
- specific surface area (BET) 35 - 65 m^2/g
- tamped density Approx. 140 g/L

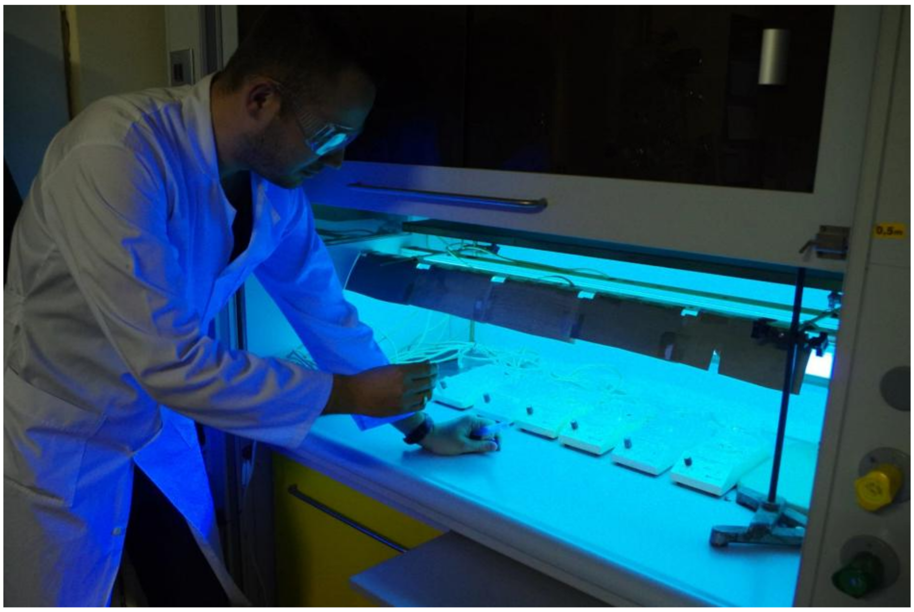

Fig. S1. The experimental stand for UV irradiation of FQs solutions.

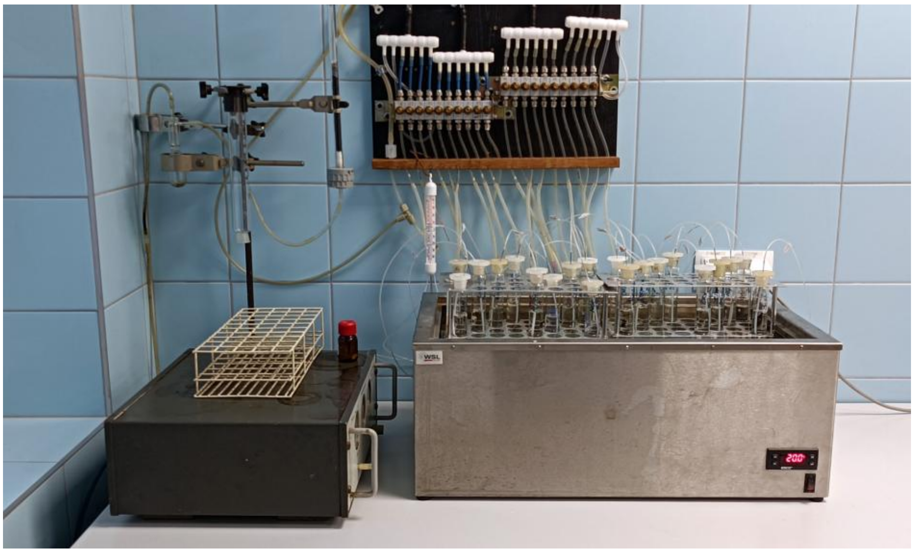

Fig. S2. The experimental stand for the biodegradation of samples under aerobic conditions.

Table S1. Characteristics of environmental samples used for conditioning solutions containing FQs and ODPs

Origin of water sample	Przemsza River	Brynica River	Source of the Sztoła River	Lake Pogoria 3
Geographic coordinates of SCI (latitude and longitude) in decimal degrees	50.259963°, 19.138610°	50.259341°, 19.136583°	50.239256°, 19.507149°	50.355522°, 19.212895
Conductivity (mS/cm)	0.725	1.671	0.574	0.723
Turbidity (FAU)	114.5±2.1	24.4±2.5	Below range	Below range
TOC (mgC/L)	29.1±3.2	13.3±0.6	2.47±0.07	5.40±0.62
Number of heterotrophic microorganisms (CFU/L)	1545±201	980±51	0	69±8

Oxygen conditions	Aerobic	Aerobic	Aerobic	Aerobic

Table S2. Ionisation conditions and acquisition parameters for the Xevo Qtof detector (XEVO G2 XS) created by the Masslynx v4.1 software

**Experimental Instrument Parameters**	
Polarity	ES+
Analyser	Resolution Mode
Capillary (kV)	3.0000
Sampling Cone	40.0000
Source Temperature (°C)	100
Source Offset	80
Desolvation Temperature (°C)	250
Cone Gas Flow (L/Hr)	50.0
Desolvation Gas Flow (L/Hr)	600.0
LM Resolution	4.7
HM Resolution	15.0
Aperture 1	0.0
Pre-filter	2.0
Ion Energy	0.2
Manual Collision Energy	FALSE
Collision Energy	6.0
Detector	2825
Sample Infusion Flow Rate (µL/min)	25
Sample Flow State	LC
**Acquisition mass range**	
Start mass	50.000
End mass	600.000
Scan Time (sec)	0.500
Interscan Time (sec)	0.014
Set Mass	Manual From Chromatogram
Start Time (mins)	0.00
End Time (mins)	10.00
Data Format	Continuum
ADC Sample Frequency (GHz)	6.0
ADC Pusher Frequency (µs)	60.0

ADC Pusher Width (μs)	1.50
Collision Energy for MSMS (eV)	10.0-35.0

1. **Assessment of the ecotoxicity of antibiotics solutions**

The MARA® microbial bioassay (NCIMB Ltd) was used in experiments to assess the ecotoxicity of antibiotic solutions. As indicator organisms, this test includes one yeast strain and eleven bacterial strains that belong to taxonomically diverse groups and that have different sensitivities to different toxicants [49]. Literature data have confirmed that the MARA® assay can be successfully applied to test the antimicrobial drug toxicity [48]. Moreover, the results of this bioassay can be considered as more representative and reliable than those obtained in single-strains tests [46-48,52]. Microorganisms were incubated in phytone peptone medium (2% w/w, Becton, Dickinson & Co.). The growth of the test microorganisms was observed as a colour change of 2,3,5-triphenyltetrazolium chloride (TZR, p.a.; POCH; Poland). The growth of test microorganisms resulted in the reduction of indicator and the formation of a coloured precipitate in the wells of plates [46-48]. The Microbial Toxic Concentration (MTC; Eq. S1) was determined using the MARA® software (NCIMB Ltd).

$$MTC = C_{\min} \times d^{(P_{tot}/P_0)-1} \quad \text{(S1)}$$

where C_{min} is the lowest concentration in the gradient, d is the dilution factor, P_o is the pellet size in the control well, and P_{tot} is the sum of the pellet sizes in all wells that were exposed to the concentration gradient of the antibiotic solutions.

Each test was performed three times. Plates were prepared according to the procedure described in Section 3, SM (Bioassay procedure) and shown in Fig. 3Sa. The initial FQs concentration was 0.1 mmol/L and the dilution step was 3.

The changes in each plate were recorded after 48 hours of incubation and analysed using MARA® software. The results were presented as means (n=3) and standard deviations of MTC values (Figs. 7 and 8 in the text).

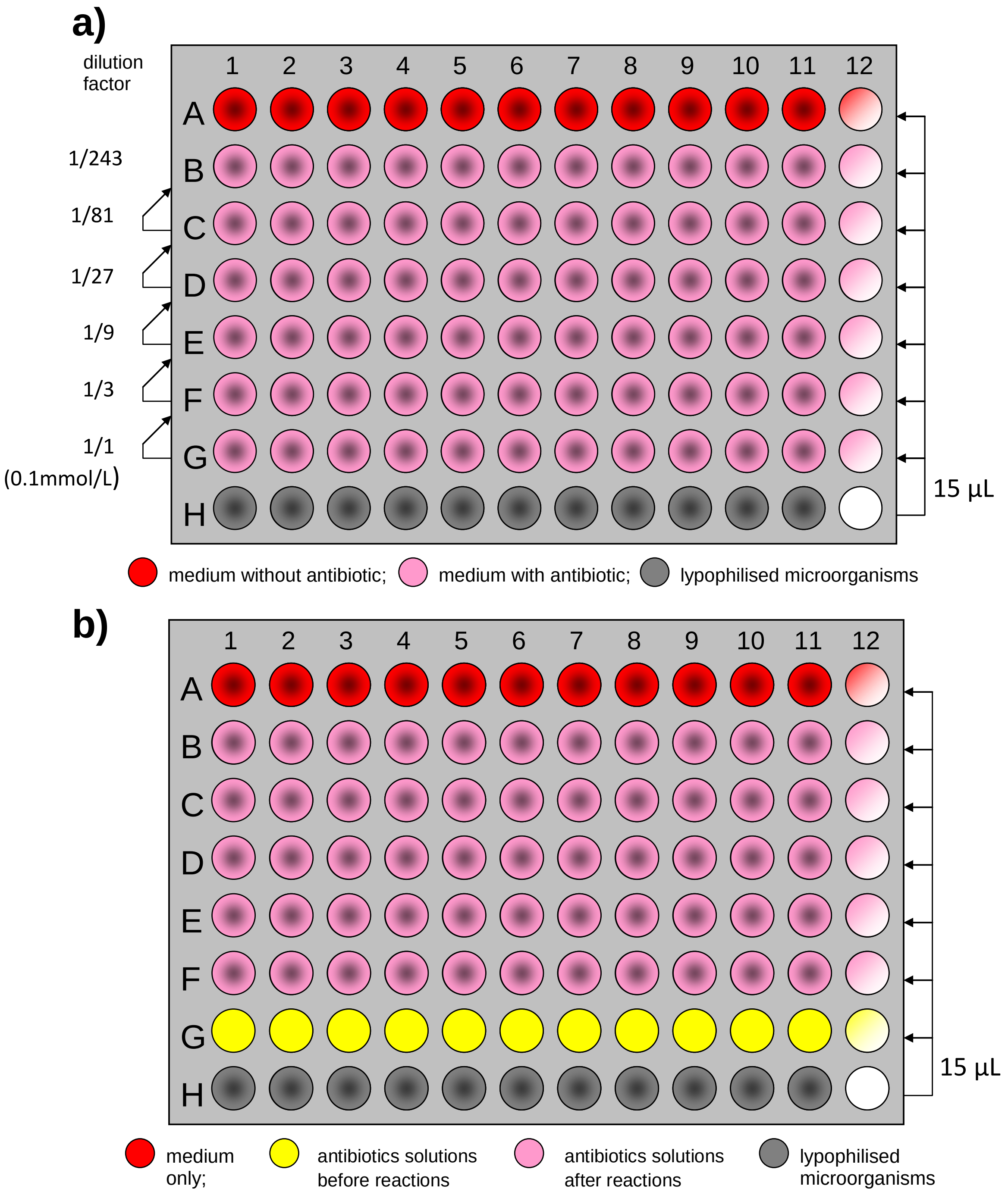

Fig. S3. Plate preparation scheme for ecotoxicity assessment of a) the antibiotics tested (determination of MTC values), b) solutions after photocatalysis/conditioning.

2. Determination of the ecotoxicity of FQs solutions before and after degradation

The antimicrobial activities of solutions containing antibiotics and their ODPs were also assessed using the MARA® assay. After degradation (photocatalysis or biodegradation), solid phytone peptone (0.4 g) and 200 µL of TZR solution (1%) were added to 10 mL of the test solutions. Then each sample was sterilised with a filter (sterile syringe filter, 28mm/0.2 µm, Minisart® NML Plus, Sartorius) and applied directly to the microplates (Fig. 3S b).

3. Bioassay procedure

Row H in columns 1-11 of the MARA® 96-well test plate contained lyophilised species: 1-*Microbacterium* spp., 2- *B. diminuta*, 3- *C. freundii*, 4- *C. testosterone*, 5- *E. casseliflavus*, 6- *D. acidovorans*, 7- *K. gibsonii*, 8- *S. warneri*, 9- *P. aurantiaca*, 10- *S. rubidaea* and 11- *P. anomalia* [5]. The column 12 was the negative control for the bioassay (without microorganisms). As a first step, 150 µL of sterile medium at a concentration of 2% (w/w) was added to each well in row H. The microplates were then incubated for 4 h at 303 K. After incubation, 100 µL of sterile medium (2% phytone peptone with 0.01% TZR) was added to each well in rows A-F. Furthermore, aliquots of 150 µL of a sterile antibiotic solution in phytone peptone medium (2%) with the addition of 0.01% TZR were placed in row G. 50 µL of solutions were transferred from each well in row G to the corresponding wells in row F. After mixing the contents of the wells, 50 µL of solution from each well in row F was transferred to the wells in row E. This procedure was repeated for row B. Row A contained only 100 µL of sterile medium, without antibiotic solution (positive control). Finally, 15 µL of inoculum was transferred from the wells in row H to the corresponding wells in rows G to A. Prepared microplates were incubated for 48 h at 303 K.

100 μL samples after photocatalytic degradation were applied to plates in rows from G to B (Fig. 3S b). Then, they were inoculated and incubated in the same way as the plates with the antibiotic.

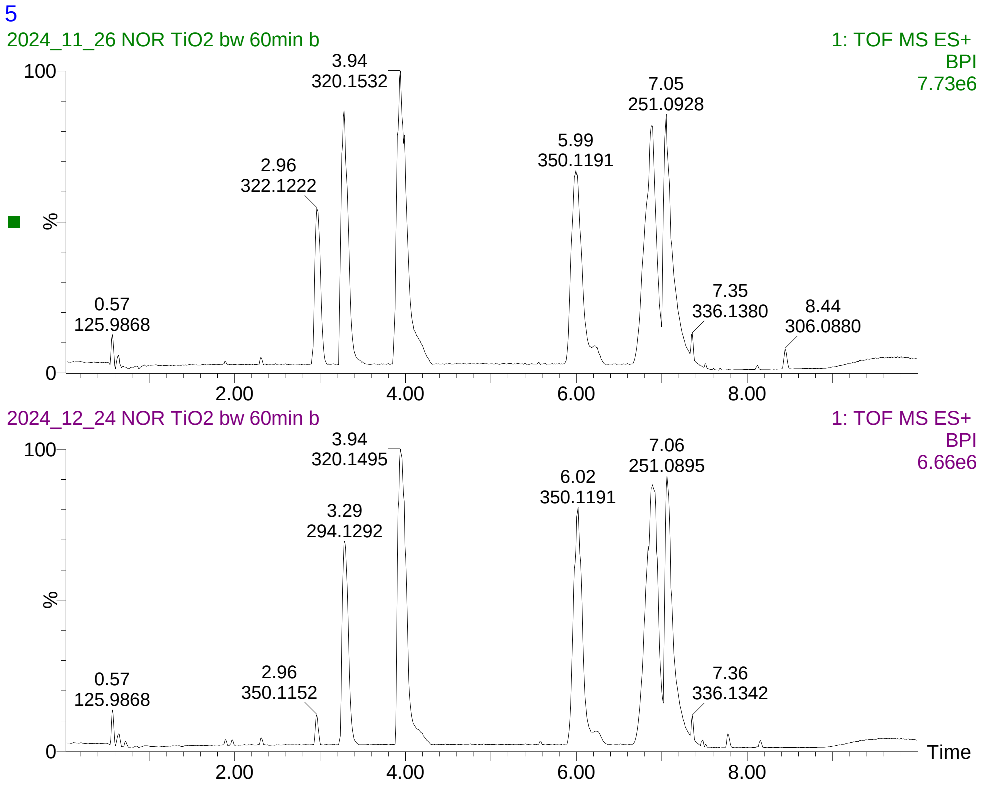

Fig. S4. Comparison of NOR solution chromatograms after photocatalytic degradation recorded at the beginning and after 28 days of conditioning in deionised water.

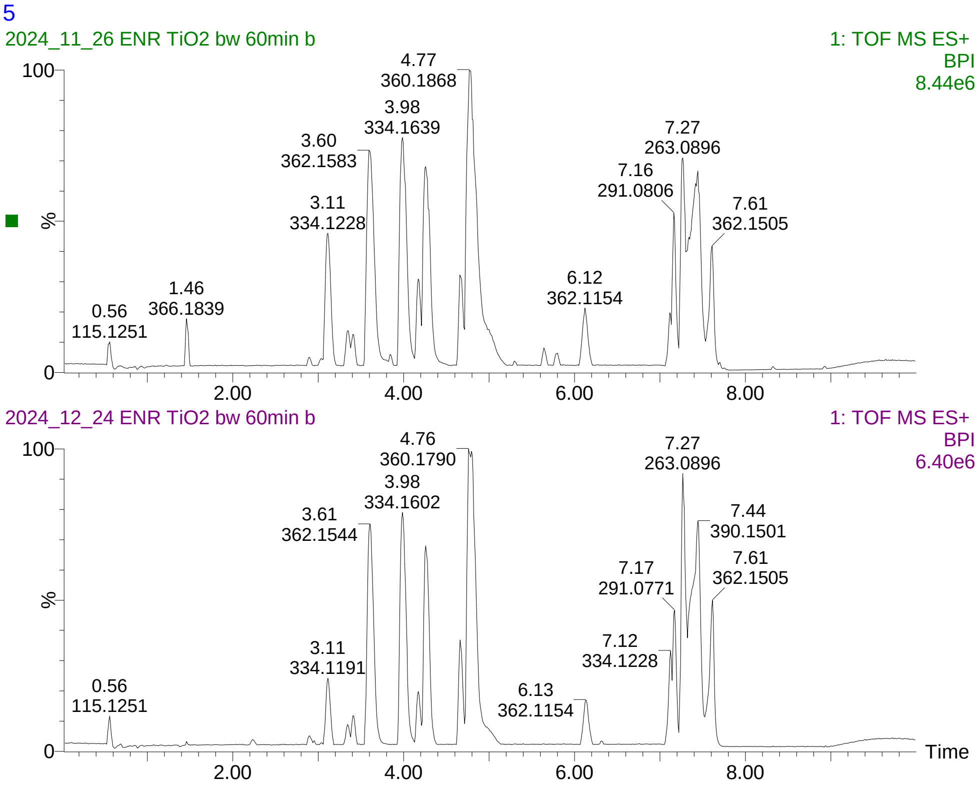

Fig. S5. Comparison of ENR solution chromatograms after photocatalytic degradation recorded at the beginning and after 28 days of conditioning in deionised water.

Table S3. Mass spectrometry data for identified ODPs after NOR degradation

Retention Time (min)	Symbol in text and in figures	m/z	Formula of parent ion	MSMS spectrum	Formula of daughter ion
1.05	PN1	298.1204	$C_{13}H_{17}N_3O_4F$	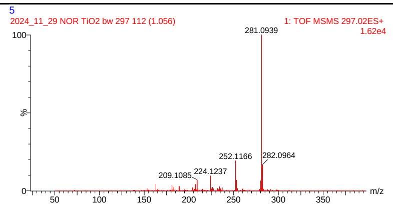	281 $C_{13}H_{14}N_2O_4F$<br>252 $C_{12}H_{15}N_3O_2F$<br>224 $C_{11}H_{15}N_3OF$<br>209 $C_{11}H_{14}N_2OF$<br>163 $C_9H_8N_2F$?
1.42	PN2	334.1203	$C_{16}H_{17}N_3O_4F$	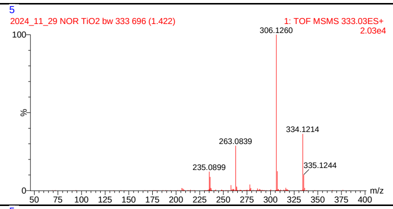	306 $C_{15}H_{17}N_3O_3F$<br>263 $C_{13}H_{12}N_2O_3F$<br>235 $C_{12}H_{12}N_2O_2F$
1.74	PN3	332.1245	$C_{16}H_{18}N_3O_5$	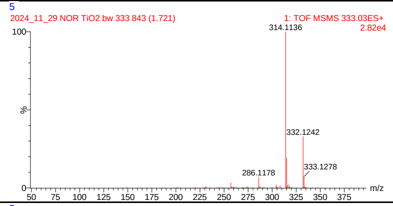	314 $C_{16}H_{16}N_3O_4$<br>286 $C_{15}H_{16}N_3O_3$<br>257 $C_{14}H_{13}N_2O_3$
1.91	PN4	296.1411	$C_{14}H_{19}N_3O_3F$	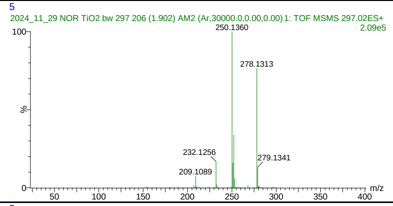	278 $C_{14}H_{17}N_3O_2F$<br>250 $C_{13}H_{17}N_3OF$<br>232 $C_{13}H_{15}N_3F$<br>209 $C_{11}H_{14}N_2OF$/$C_{14}H_{13}N_2$
1.99	PN5	270.1255	$C_{12}H_{17}N_3O_3F$	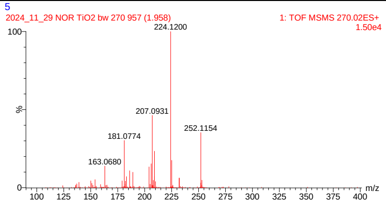	252 $C_{12}H_{15}N_3O_2F$<br>224 $C_{11}H_{15}N_3OF$<br>207 $C_{11}H_{12}N_2OF$<br>181 $C_9H_{10}N_2OF$<br>163 $C_9H_8N_2F$

2.34	PN6	334.1207	$C_{16}H_{17}N_3O_4F$	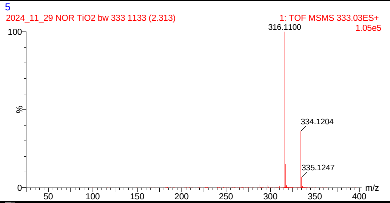	316 $C_{16}H_{15}N_3O_3F$<br>296 $C_{13}H_{15}N_3O_4F$<br>288 $C_{15}H_{15}N_3O_2F$
2.50	PN7	308.1051	$C_{14}H_{15}N_3O_4F$	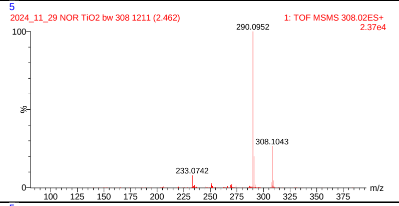	290 $C_{14}H_{13}N_3O_3F$<br>233 $C_{12}H_{10}N_2O_2F$
2.60	PN8	318.1456	$C_{16}H_{20}N_3O_4$<br>/$C_{13}H_{21}N_3O_5F$	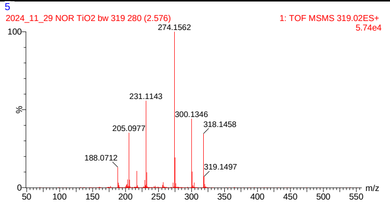	300 $C_{16}H_{18}N_3O_3$ /$C_{13}H_{19}N_3O_4F$<br>274 $C_{15}H_{20}N_3O_2$ /$C_{12}H_{21}N_3O_3F$<br>231 $C_{13}H_{15}N_2O_2$ /$C_{10}H_{16}N_2O_3F$<br>205 $C_{11}H_{13}N_2O_2$ /$C_8H_{14}N_2O_3F$<br>188 $C_{11}H_{10}NO_2$/ $C_8H_{11}NO_3F$
2.86	PN9	326.1154	$C_{14}H_{17}N_3O_5F$	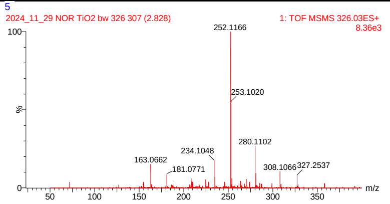	308 $C_{14}H_{15}N_3O_4F$<br>280 $C_{13}H_{15}N_3O_3F$<br>252 $C_{12}H_{15}N_3O_2F$<br>234 $C_{12}H_{13}N_3OF$<br>181 $C_9H_{10}N_2OF$<br>163 $C_9H_8N_2F$
2.98	PN10	322.1210	$C_{15}H_{17}N_3O_4F$	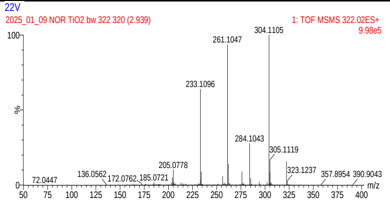	304 $C_{15}H_{15}N_3O_3F$<br>284 $C_{15}H_{14}N_3O_3$ /$C_{12}H_{15}N_3O_4F$<br>261 $C_{14}H_{14}N_2O_2F$<br>233 $C_{13}H_{14}N_2OF$<br>205 $C_{11}H_{10}N_2OF$

3.16	PN11	280.1461	$C_{14}H_{19}N_3O_2F$	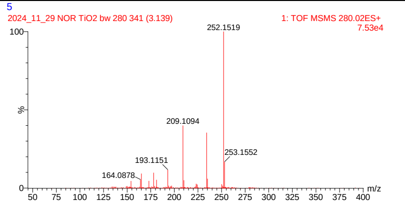	252 $C_{13}H_{19}N_3OF$<br>234 $C_{13}H_{17}N_3F$<br>209 $C_{11}H_{14}N_2OF$ /$C_{14}H_{13}N_2$<br>193 $C_{11}H_{14}N_2F$<br>178 $C_{10}H_{11}N_2F$<br>165 $C_9H_{10}N_2F$
3.26	PN12	294.1259	$C_{14}H_{17}N_3O_3F$	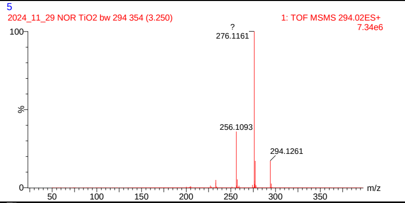	276 $C_{14}H_{15}N_3O_2F$<br>256 $C_{11}H_{15}N_3O_3F$ /$C_{14}H_{14}N_3O_2$<br>233 $C_{13}H_{14}N_2OF$
3.50	PN13	334.1399	$C_{16}H_{20}N_3O_5$ /$C_{13}H_{21}N_3O_6F$	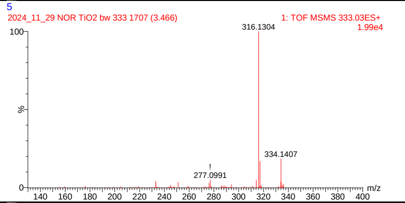	316 $C_{16}H_{18}N_3O_4$ /$C_{13}H_{19}N_3O_5F$<br>251 $C_{15}H_{11}N_2O_2$ /$C_{12}H_{12}N_2O_3F$<br>233 $C_{13}H_{14}N_2OF$?<br>176 ?
3.97	NOR	320.1411 **NOR**	$C_{16}H_{19}N_3O_3F$	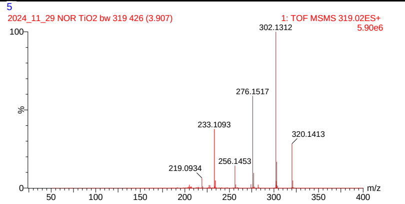	302 $C_{16}H_{17}N_3O_2F$<br>276 $C_{15}H_{19}N_3OF$<br>256 $C_{15}H_{18}N_3O$<br>233 $C_{13}H_{14}N_2OF$<br>219 $C_{12}H_{12}N_2OF$
4.48	PN14	348.1195	$C_{16}H_{18}N_3O_6$	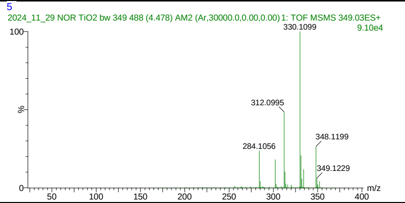	330 $C_{16}H_{16}N_3O_5$<br>312 $C_{16}H_{14}N_3O_4$<br>302 $C_{15}H_{16}N_3O_4$<br>284 $C_{15}H_{14}N_3O_3$

4.85	PN15	249.0875	$C_{12}H_{13}N_2O_4$	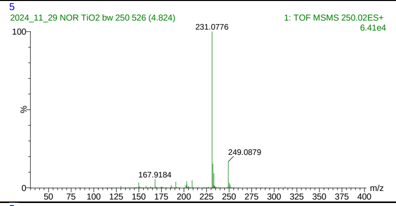	231 $C_{12}H_{11}N_2O_3$<br>203 $C_{10}H_7N_2O_3$
5.18	PN16	322.0845	$C_{14}H_{13}N_3O_5F$	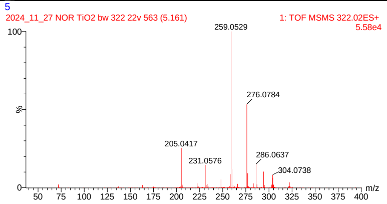	304 $C_{14}H_{11}N_3O_4F$<br>294 $C_{13}H_{13}N_3O_4F$<br>286 $C_{14}H_9N_3O_3F$<br>276 $C_{13}H_{11}N_3O_3F$<br>231 $C_{12}H_8N_2O_2F$<br>205 $C_{10}H_6N_2O_2F$
5.35	PN17	251.0471	$C_{11}H_8N_2O_4F$	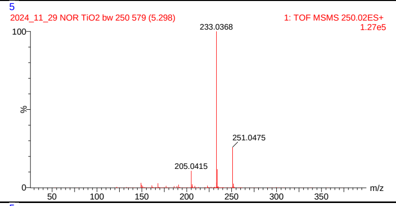	233 $C_{11}H_6N_2O_3F$<br>205 $C_{10}H_6N_2O_2F$<br>149 $C_8H_6N_2F$
5.48	PN18	348.0996	$C_{16}H_{15}N_3O_5F$	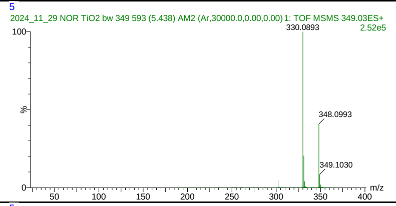	330 $C_{16}H_{13}N_3O_4F$<br>302 $C_{14}H_9N_3O_4F$
5.59	PN19	336.0996	$C_{15}H_{15}N_3O_5F$	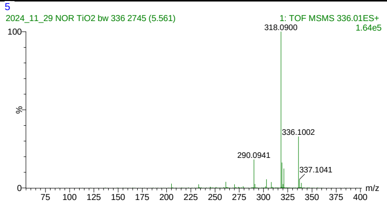	318 $C_{15}H_{13}N_3O_4F$<br>303 $C_{15}H_{12}N_2O_4F$<br>290 $C_{14}H_{13}N_3O_3F$<br>205 $C_{10}H_6N_2O_2F$

6.04	PN20	350.1152	$C_{16}H_{17}N_3O_5F$	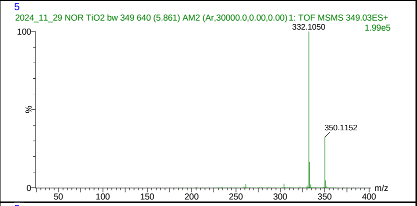	332 $C_{16}H_{15}N_3O_4F$<br>304 $C_{15}H_{15}N_3O_3F$<br>261 $C_{13}H_{10}N_2O_3F$
6.92	PN21	322.1207	$C_{15}H_{17}N_3O_4F$	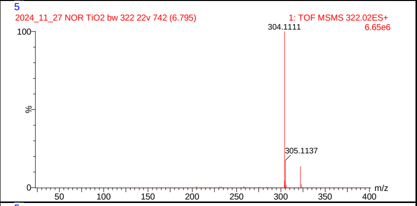	304 $C_{15}H_{15}N_3O_3F$
7.08	PN22	251.0835	$C_{12}H_{12}N_2O_3F$	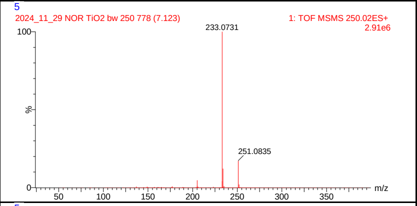	233 $C_{12}H_{10}N_2O_2F$<br>205 $C_{10}H_6N_2O_2F$
7.36	PN23	336.1363	$C_{16}H_{19}N_3O_4F$	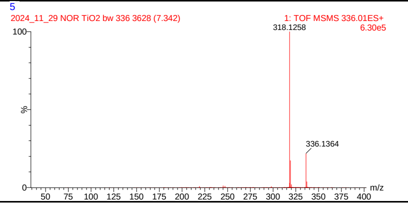	318 $C_{16}H_{17}N_3O_3F$<br>219 $C_{11}H_8N_2O_2F$
7.62	PN24	290.0943	$C_{14}H_{13}N_3O_3F$	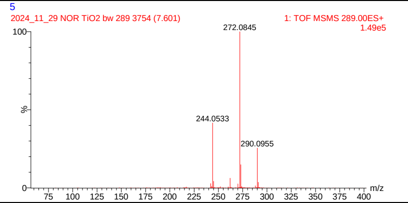	272 $C_{14}H_{11}N_3O_2F$<br>262 $C_{12}H_9N_3O_3F$?<br>244 $C_{12}H_7N_3O_2F$

Table S4. Mass spectrometry data for identified ODPs after ENR degradation

Retention Time (min)	Symbol in text and in figures	m/z	Formula of parent ion	MSMS spectrum	Formula of daughter ion
1.00	PE1	340.1664	$C_{19}H_{22}N_3O_3$	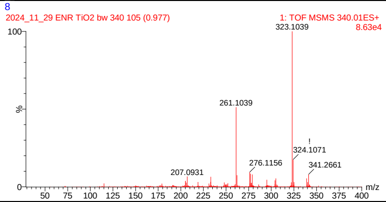	323 $C_{18}H_{15}N_2O_4$<br>261 $C_{17}H_{13}N_2O$<br>233 $C_{16}H_{13}N_2$<br>207 $C_{14}H_{11}N_2$
1.20	PE2	368.1613	$C_{17}H_{23}N_3O_5F$	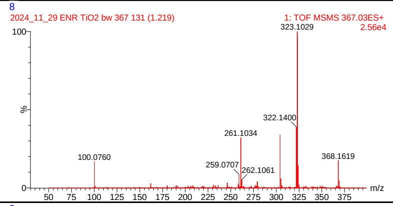	323 $C_{15}H_{16}N_2O_5F$<br>304 $C_{12}H_{19}N_3O_5F$<br>261 $C_{14}H_{14}N_2O_2F$<br>100 $C_5H_{10}NO$
1.37	PE3	376.1502	$C_{18}H_{22}N_3O_6$	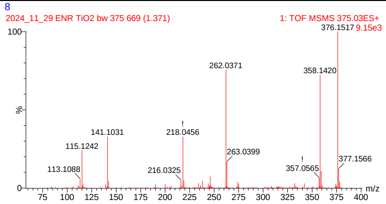	358 $C_{18}H_{20}N_3O_5$<br>262 $C_{15}H_6N_2O_3$<br>218 $C_{11}H_8NO_4$<br>141 $C_7H_{13}N_2O$
1.46	PE4	366.1825	$C_{18}H_{25}N_3O_4F$	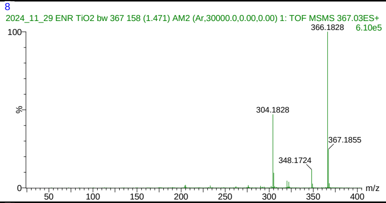	348 $C_{18}H_{23}N_3O_3F$<br>304 $C_{17}H_{23}N_3OF$?
1.61	PE5	338.1516	$C_{16}H_{21}N_3O_4F$	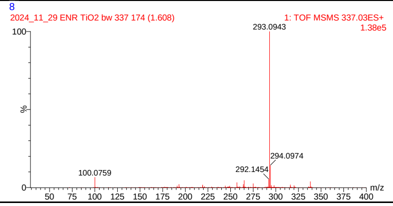	293 $C_{14}H_{14}N_2O_4F$<br>100 $C_5H_{10}NO$

1.80	PE6	364.1671	$C_{18}H_{23}N_3O_4F$	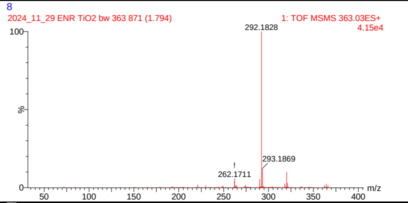	320 $C_{17}H_{23}N_3O_2F$<br>292 $C_{16}H_{23}N_3OF$<br>262 $C_{15}H_{21}N_3F$?
2.01	PE7	390.1465	$C_{19}H_{21}N_3O_5F$	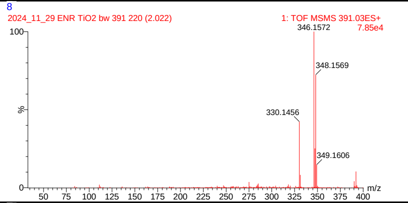	348 $C_{17}H_{22}N_3O_5$<br>346 $C_{18}H_{21}N_3O_3F$<br>330 $C_{17}H_{20}N_4O_4$ /$C_{14}H_{21}N_3O_5F$
2.25	PE8	334.1567	$C_{17}H_{21}N_3O_3F$	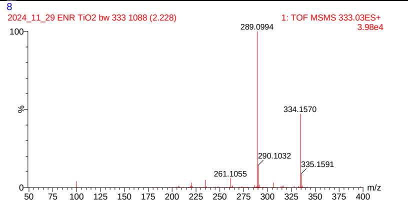	289 $C_{15}H_{14}N_2O_3F$<br>261 $C_{14}H_{14}N_2O_2F$<br>235 $C_{12}H_{12}N_2O_2F$<br>100 $C_5H_{10}NO$
2.56	PE9	340.1674	$C_{16}H_{23}N_3O_4F$	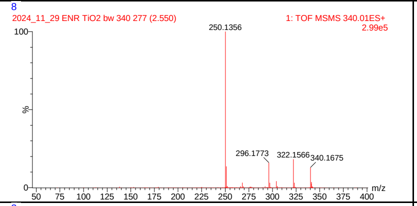	322 $C_{16}H_{21}N_3O_3F$<br>304 $C_{16}H_{19}N_3O_2F$<br>296 $C_{15}H_{23}N_3O_2F$<br>250 $C_{13}H_{17}N_3OF$
2.72	PE10	320.1047	$C_{15}H_{15}N_3O_4F$	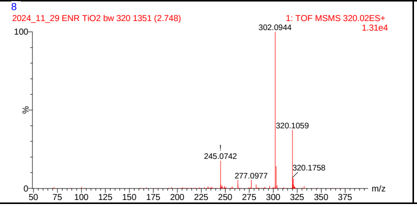	302 $C_{15}H_{13}N_3O_3F$<br>245 $C_{13}H_{10}N_2O_2F$

2.90	PE11	336.1723	$C_{17}H_{23}N_3O_3F$	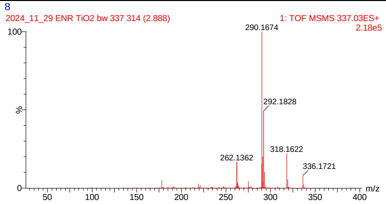	318 $C_{17}H_{21}N_3O_2F$<br>290 $C_{16}H_{21}N_3OF$<br>262 $C_{14}H_{17}N_3OF$?<br>262 $C_{15}H_{21}N_3F$?<br>178 $C_{10}H_9NOF$
3.04	PE12	360.1563	$C_{18}H_{22}N_3O_5$<br>/$C_{15}H_{23}N_3O_6F$	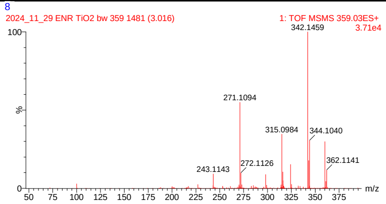	344 $C_{17}H_{15}N_3O_4F$?<br>342 $C_{18}H_{20}N_4O_4$ /$C_{15}H_{21}N_3O_5F$<br>324 $C_{18}H_{18}N_3O_3$ /$C_{15}H_{19}N_3O_4F$<br>315 $C_{16}H_{15}N_2O_5$ /$C_{13}H_{16}N_2O_6F$<br>271 $C_{15}H_{15}N_2O_3$ /$C_{12}H_{16}N_2O_4F$<br>243 $C_{11}H_{16}N_2O_3F$<br>100 $C_5H_{10}NO$
3.11	PE13	334.1205	$C_{19}H_{17}N_3O_4F$	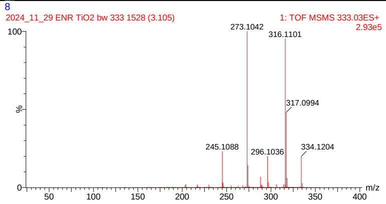	316 $C_{16}H_{15}N_3O_3F$<br>296 $C_{16}H_{14}N_3O_3$/<br>$C_{13}H_{15}N_3O_4F$<br>273 $C_{15}H_{14}N_2O_2F$<br>245 $C_{14}H_{14}N_2OF$
3.34	PE14	344.1411	$C_{18}H_{19}N_3O_3F$	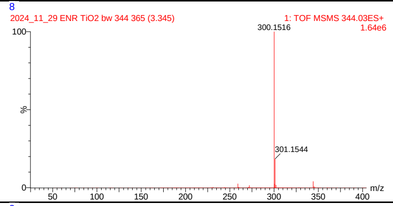	300 $C_{17}H_{19}N_3OF$<br>259 $C_{14}H_{14}N_3OF$
3.42	PE15	358.1767	$C_{19}H_{24}N_3O_4$	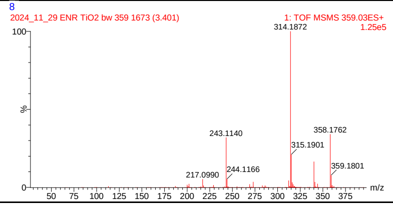	340 $C_{19}H_{22}N_3O_3$ /$C_{16}H_{23}N_3O_4F$<br>314 $C_{18}H_{24}N_3O_2$<br>243 $C_{14}H_{15}N_2O_2$<br>217 $C_{12}H_{13}N_2O_2$

3.60	PE16	362.1516	$C_{18}H_{21}N_3O_4F$	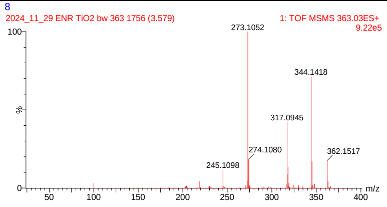	344 $C_{18}H_{19}N_3O_3F$<br>317 $C_{16}H_{14}N_2O_4F$<br>273 $C_{15}H_{14}N_2O_2F$<br>245 $C_{14}H_{14}N_2OF$<br>100 $C_5H_{10}NO$?
3.85	PE17	390.1457	$C_{19}H_{21}N_3O_5F$	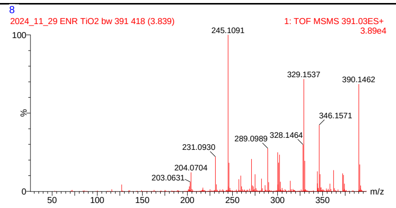	362 $C_{17}H_{17}N_3O_5F$<br>346 $C_{18}H_{21}N_3O_3F$<br>329 $C_{18}H_{20}N_3O_2F$<br>300 $C_{17}H_{19}N_3OF$<br>289 $C_{15}H_{14}N_2O_3F$<br>245 $C_{14}H_{14}N_2OF$<br>231 $C_{13}H_{12}N_2OF$<br>204 $C_{11}H_9N_2OF$<br>127 $C_6H_{11}N_2O$
3.93	PE18	334.1570	$C_{17}H_{21}N_3O_3F$	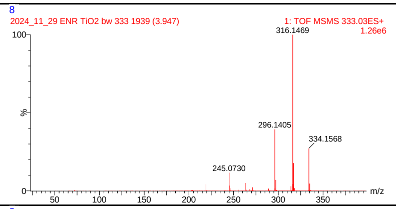	316 $C_{17}H_{19}N_3O_2F$<br>296 $C_{14}H_{19}N_3O_3F$ / $C_{17}H_{18}N_3O_2$<br>245 $C_{13}H_{10}N_2O_2F$<br>219 $C_{12}H_{12}N_2OF$
4.18	PE19	332.1413	$C_{17}H_{19}N_3O_3F$	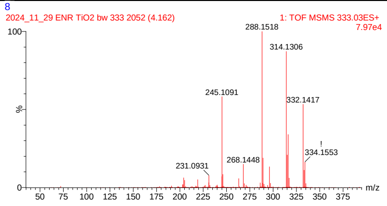	314 $C_{17}H_{17}N_3O_2F$<br>288 $C_{16}H_{19}N_3OF$<br>268 $C_{16}H_{18}N_3O$?<br>245 $C_{14}H_{14}N_2OF$<br>231 $C_{13}H_{12}N_2OF$

4.26	PE20	358.1564	$C_{19}H_{21}N_3O_3F$	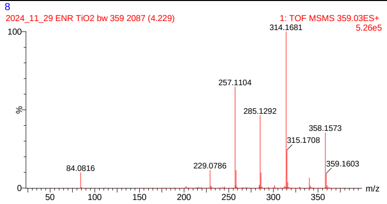	340 $C_{19}H_{19}N_3O_2F$<br>314 $C_{18}H_{21}N_3OF$?<br>285 $C_{16}H_{16}N_3OF$<br>257 $C_{15}H_{14}N_2OF$<br>229 $C_{13}H_{10}N_2OF$<br>84 $C_5H_{10}N$
4.68	PE21	376.1673	$C_{19}H_{23}N_3O_4F$	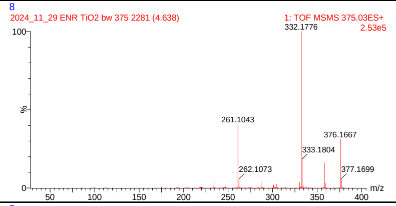	358 $C_{19}H_{21}N_3O_3F$<br>332 $C_{18}H_{23}N_3O_2F$<br>261 $C_{14}H_{14}N_2O_2F$
4.78	ENR	360.1752<br>**ENR**	$C_{19}H_{23}N_3O_3F$	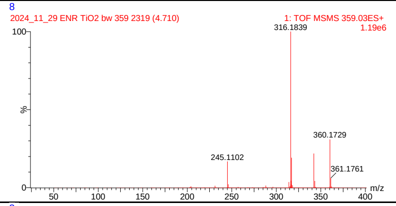	342 $C_{19}H_{21}N_3O_2F$<br>316 $C_{18}H_{23}N_3OF$<br>245 $C_{14}H_{14}N_2OF$
5.29	PE22	376.1670	$C_{19}H_{23}N_3O_4F$	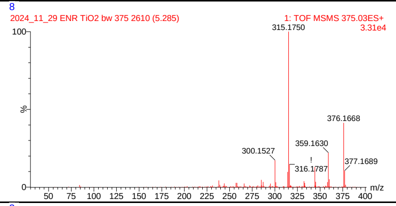	359 $C_{19}H_{22}N_3O_3F$<br>344 $C_{18}H_{19}N_3O_3F$<br>315 $C_{18}H_{22}N_3OF$<br>300 $C_{17}H_{19}N_3OF$
5.67	PE23	392.1614	$C_{19}H_{23}N_3O_5F$	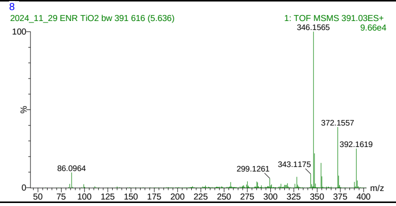	372 $C_{19}H_{22}N_3O_5$<br>354 $C_{19}H_{20}N_3O_4$ /$C_{16}H_{21}N_3O_5F$<br>346 $C_{18}H_{21}N_3O_3F$<br>89 $C_5H_{12}N$

5.78	PE24	374.1511	$C_{19}H_{21}N_3O_4F$	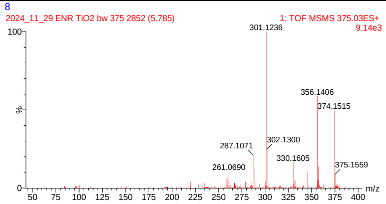	356 $C_{19}H_{19}N_3O_3F$<br>330 $C_{18}H_{21}N_3O_2F$<br>301 $C_{16}H_{16}N_3O_2F$<br>287 $C_{15}H_{14}N_3O_2F$<br>261 $C_{13}H_{10}N_2O_3F$
6.12	PE25	362.1151	$C_{17}H_{17}N_3O_5F$	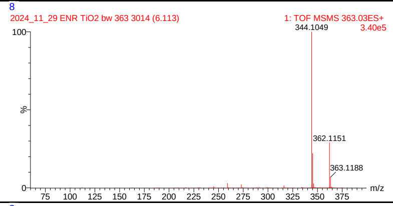	344 $C_{17}H_{15}N_3O_4F$
7.12	PE26	334.1206	$C_{16}H_{17}N_3O_4F$	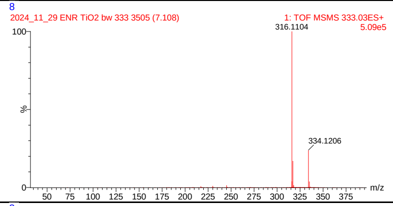	316 $C_{16}H_{15}N_3O_3F$
7.50	PE27	390.1467	$C_{19}H_{21}N_3O_5F$	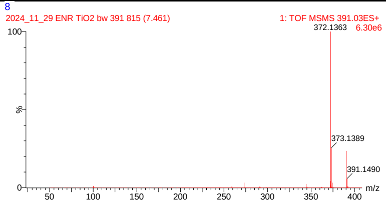	372 $C_{19}H_{19}N_3O_4F$<br>344 $C_{18}H_{19}N_3O_3F$<br>273 $C_{14}H_{10}N_2O_3F$<br>100 $C_5H_{10}NO$
